# *FC_analysis*: a tool for investigating atomic force microscopy maps of force curves

**DOI:** 10.1186/s12859-018-2265-4

**Published:** 2018-07-06

**Authors:** Simone Dinarelli, Marco Girasole, Giovanni Longo

**Affiliations:** Istituto di Struttura della Materia, CNR, Rome, Italy

**Keywords:** AFM, Automated analysis, Force curves, Force volume, Elasticity, Stiffness, Erythrocytes, Meteorites, Tissues

## Abstract

**Background:**

The collection and analysis of Atomic Force Microscopy force curves is a well-established procedure to obtain high-resolution information of non-topographic data from any kind of sample, including biological specimens. In particular, these analyses are commonly employed to study elasticity, stiffness or adhesion properties of the samples. Furthermore, the collection of several force curves over an extended area of the specimens allows reconstructing maps, called force volume maps, of the spatial distribution of the mechanical properties. Coupling these maps with the conventional high-resolution topographic reconstruction of the sample’s surface, provides a deeper insight on the sample composition from the structural and nanomechanical point of view.

**Results:**

In this paper we present the open source software package *FC_analysis* that automatically analyses single force curves or entire force volume maps to yield the corresponding elasticity and deformability images. The principal characteristic of the *FC_analysis* is a large adaptability to the various experimental setups and to different analysis methodologies. For instance, the user can provide custom values for the detector sensitivity, scanner-z sensitivity, cantilever’s elastic constant and map’s acquisition modality and can choose between different analysis methodologies. Furthermore, the software allows the optimization of the fitting parameters and gives direct control on each step of the analysis procedure. Notably, to overcome a limitation common to many other analysis programs, *FC_analysis* can be applied to a rectangular portion of the image, allowing the analysis of inhomogeneous samples. Finally, the software allows reconstructing a Young’s modulus map at different penetration depths, enabling the use of modern investigation tools such as the force tomography.

**Conclusions:**

The *FC_analysis* software aims to become a useful tool for the analysis of force curves maps collected using custom or commercial Atomic Force Microscopes, and is especially useful in those cases for which the producer doesn’t release a dedicated software.

**Electronic supplementary material:**

The online version of this article (10.1186/s12859-018-2265-4) contains supplementary material, which is available to authorized users.

## Background

An Atomic Force Microscope (AFM) consists of a sharp tip, with radius of curvature in the nanometer range, attached to the end of a flexible cantilever with a reflective coverage on the back and a measurable elastic constant. In the most common set-up, a laser is focused on the back of the cantilever and the reflected spot is usually monitored using a 4-quadrant detector that allows a sensitive evaluation of the tip position and of the cantilever deflection. An xyz stage, generally made of piezoelectric materials, is driven by a feedback loop in order to scan the xy plane of a sample surface while maintaining a constant tip-sample distance in the z direction or interaction. The scan results in an image that is intrinsically tridimensional and quantitative [1].

In addition to reconstructing the topography of the sample, the AFM tip can be used to measure at the nanoscale additional sample properties, such as elasticity, hardness or adhesion. To perform these kind of measurements, the tip is pushed forward on the samples until a maximum predetermined interaction (load) is reached and then is retracted. The collection of the cantilever deflection during this procedure is used to build a force curve (FC), which provides information on the local mechanical properties of the sample [2]. For instance, in “soft” samples, such as the majority of biological specimens, the nanomechanical behaviour is dominated by the elasticity. As consequence, by comparing the tip-sample interaction curve measured on a soft sample with that collected on undeformable surface, we can reconstruct an indentation curve, as function of the applied load. By fitting the values of this curve using different models, we can quantitatively determine the stiffness of the area under investigation.

The capability to measure the nanomechanical properties of samples has pushed forward the potential of AFM in different research fields, ranging from materials science to biology. Concerning these latter applications, in particular, the AFM is a very effective technique as it can work, without loss of sensibility, in liquid physiological environment. Liquid AFM allows the characterization of soft biological samples in native conditions and can even be used to monitor the evolution of living cells. The resulting combination of nanometer-resolution morphological images with local measurements of the sample’s elastic properties makes the AFM a very popular instrument for biophysical and cellular biology studies and a powerful diagnostic tool in biomedical applications. Indeed, correlating different information from the very same area allows a unique characterization of the samples: for instance, a correlation between cell’s mechanical properties and functionality has already been proven in many bio-systems [3–5] with consequences in both physiological and pathological contexts.

The improvement in the acquisition performances of the AFM make nowadays possible to collect high resolution force volume (FV) maps (256 × 256 points, or more) in a time not far from the standard imaging mode. However, the collected FV maps must be post-processed and the analysis of such a large amount of data requires a consistent and automatized system. Furthermore, despite the existence of several different open or dedicated software tools to study FV maps, most of them share a common problem because, as stated by Lin and co. [6], in a given map individual force curves can have very different shapes, requiring different analytical approaches to obtain consistent quantitative nanomechanical results from the raw data.

The parameters, the routines and the methods that are commonly used to analyse the FCs often depend from the experimental setup, the microscope specifications and the adopted approximations. As consequence, the routines developed by the software present on the market are often scarcely portable among microscopes and laboratories. This contributes to the large experimental variability of literature results from different groups, which are not easily compared quantitatively.

Following the many papers devoted to the analytical discussion of the best methodology to analyse a FC according to a particular experimental context [7–9], we propose the *FC_analysis* package as a step forward towards the solution to the problem of FC and large FV analysis. This free and open software tool has an explicit, simple and direct access and gives the user complete control over the most important parameters of the experimental setup and the most appropriate theoretical model for the chosen experimental conditions.

*FC_analysis* has several advantages over the available software to manage force volume data. As an example, OpenFovea [10] can’t be used to process force curves in .txt form (the routine is currently not implemented and there are no updates from 2012). In a second example, the Matlab-based software FRAME [11] is optimized for force maps acquired using the Asylum Research AFMs and requires the entire Matlab program, version 2014b or newer, to be employed.

This software package aims to become a useful tool for the analysis of FC maps collected using custom or commercial AFMs, and is especially useful in those cases for which the producer doesn’t release a dedicated software of analysis. It is important to observe that, in some cases, the AFM producers employ proprietary software that create FC maps with specific “closed” format (e.g. JPK, Bruker, Park, Nanosurf etc.).

## Implementation

*FC_analysis* is a windows executable file, without copyright, provided as additional file (see Additional file 1).

It requires the latest Matlab Compiler Runtime (MCR) (version 8.0 or newer) that can be freely downloaded from the Mathworks website: http://uk.mathworks.com/products/compiler/mcr.

**Fig. 1:**
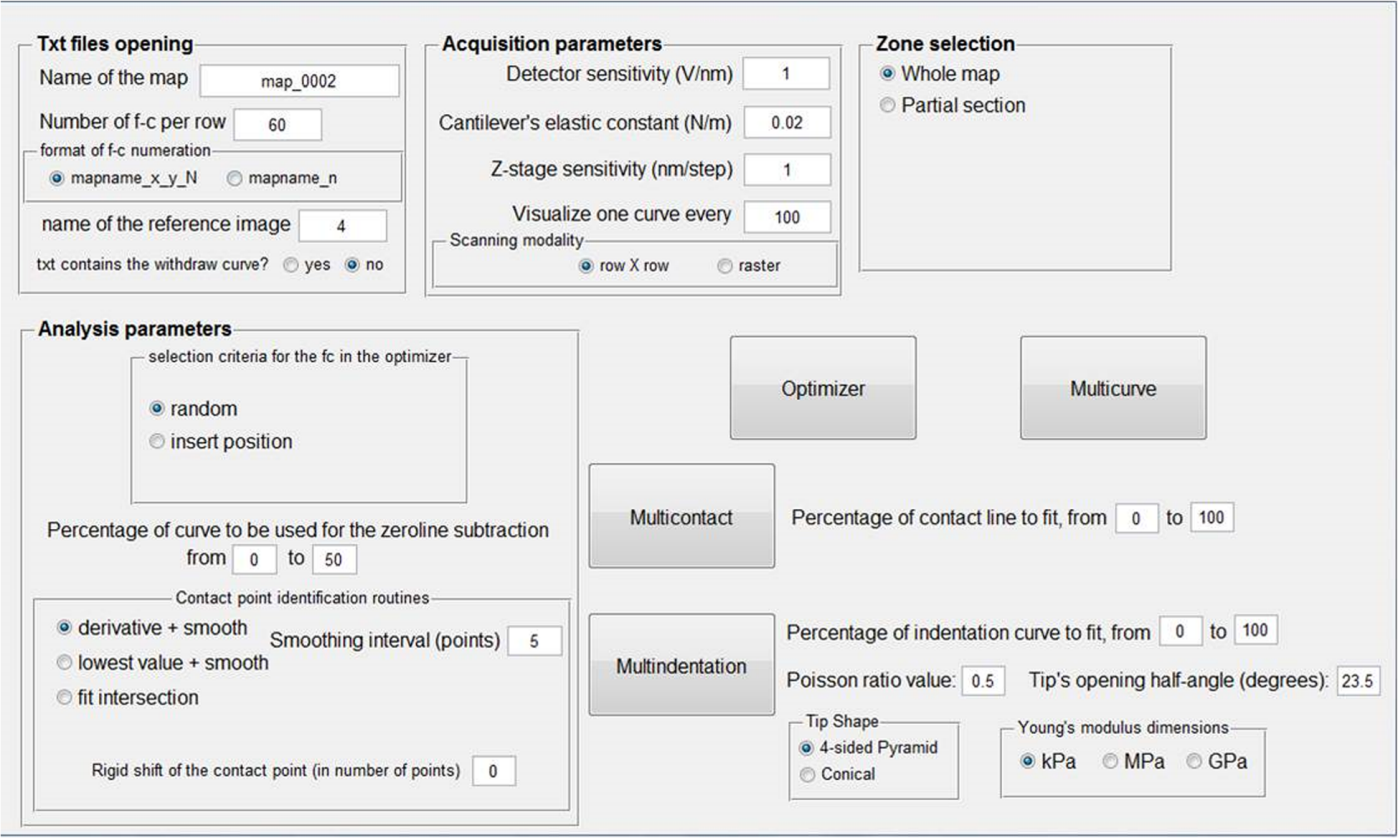
Guided User Interface (GUI) of the *FC_analysis* software. The four boxes highlight the sections in which the parameters can be changed: Txt files opening, Acquisition parameters, Zone selection and Analysis parameters. The four buttons launch the analysis routines: *Optimizer*, *Multicurve*, *Multicontact*, *Multindentation*

The software package contains four different analysis routines: *Optimizer*, *Multicurve*, *Multicontact* and *Multindentation*. The *FC_analysis* Guided User Interface (GUI), reported in Fig. 1, is divided in several modules (sections): *Txt files opening*, *Acquisition parameters*, *Zone selection* and *Analysis parameters*.

### Txt files opening

A preliminary requirement is that the FC maps need to be a collection of *.txt files, to this end, in the case of commercial AFMs, it is necessary an additional “format transducer” software, whose characteristics will obviously depend on the commercial AFM under consideration (including release and upgrades of the acquisition software), that extract the single FC from the map. The *FC_analysis* executable needs to be copied into the folder that contains the FCs. It requires that each txt file contains a single FC, reported as Z-displacement versus deflection signal. The FC can be either complete (approach and withdraw) or partial (e.g. only the approach part), and in this section one of these two cases can be selected. If the curve is complete, the withdrawal part will be automatically removed by a preliminary cut of the txt file by following the Z-displacement axis. The deflection axis has to be expressed in volts (V) or Newtons (N), the operator has to provide the number of FCs per row.

*FC_analysis* accepts two different naming modes:

A) explicit: “mapname(posx)_(posy)_(name of reference image)”
B) progressive: “mapname(progressive number)”

An example for the first case could be a FV map composed by files named “map01_32_15_img4”: map01_ is the “mapname” prefix common to all the files, the numbers 32 and 15 indicate the force curve in position 32 on x and 15 on y; img4 is the name of the reference image. The underscores are used to distinguish between x, y position and the name of the reference image.

An example for the second case could be the map composed by files named “map01_33”: map01_ is the “mapname” prefix while the number 33 means the 33th force curve of the map. The main difference between the two naming modes resides in the determination of the FC position in the map: in the first case, this position is explicit, in the second it depends on the scanning modality (see next paragraph) and on the number of FCs per row.

### Acquisition parameters

This section discusses the management of the experimental parameters and the visualization of the analysed curves.

The main parameters to control the visualization of the curves are:

- *Z-stage sensitivity*: this parameter converts the Z-stage position from the specific output of the microscope software to nanometres (nm). As an example, if the microscope yields the Z-position values in micrometres (μm), the Z-stage sensitivity must be set to 1000. In some cases, the microscope produces the Z-values expressed in piezo stage steps. In this case, the correct parameter will be the calibrated nm per step value.
- *Cantilever’s elastic constant (K*_*C*_): the exact value of the elastic constant of the cantilever used in the experiment, expressed Newtons per meter (N/m). Typically, this value is calculated from the geometric properties of the cantilever or by measuring the resonant frequency of the sensor.
- *Detector sensitivity (S)*: this parameter depends on the detector and on the optical alignment. To calculate the sensitivity, the user must collect a FC on a hard, uncompressible, surface and calculate the slope (measured in V/nm) of the approach section of the curve.

These parameters are combined to transform the cantilever’s deflection signal (D), expressed in volts, into a force between tip and sample (F), expressed in nano-Newtons (nN), through the formula (1):

1 $$ F=D\frac{K_C}{S} $$

This means that if the ordinates of the FCs are already expressed in nN, instead of V, the values of K_C_ and S have both to be set equal to 1.

In addition to these parameters, the software allows the user control over:

- *Visualize one curve every*: allows defining how many analysed FCs will be shown in real time, in a dedicated window. The default value is 100, meaning that one curve every 100 will be shown.
- *Scanning modality*: this parameter is dedicated to the automatized analysis of force volume images. The software can work with two different X/Y scanning modalities: “*raster scan*” and “*row x row*”. The raster scan modality consists in a continuous scanning, resulting in odd lines acquired from left to right and even lines acquired from right to left. In the row x row modality, every row of the image is measured by moving always from left to right. This choice is important to match the microscope’s measurement pattern and is needed if the “*progressive*” naming modality is active.

### Zone selection

This section allows the selection of the part of the map to be analysed.

The default option is set to “*whole map*”: in this condition, the analysis will be carried out on the entire set of FCs. By selecting “*partial section*” and inserting the position X and Y of the starting (bottom left corner) and ending (upper right corner) points, the user can perform the analysis only on a rectangular portion of the map. This choice influences all the analysis routines that will be used.

### Analysis parameters

The *Analysis Parameters* module allows controlling all the parameters involved in the preliminary analysis of the FCs. It is composed of 3 subsections called, respectively, “*selection criteria*”, “p*ercentage of curve used for the zeroline subtraction*” and “*contact point identification*”.

**Fig. 2:**
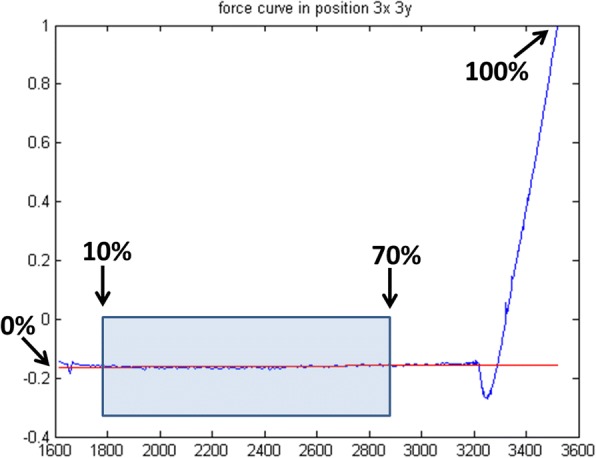
Example of zeroline subtraction fit, the linear fit (red line) is performed considering the points in the interval 10–70% of the curve (blue-highlighted section)

**Fig. 3:**
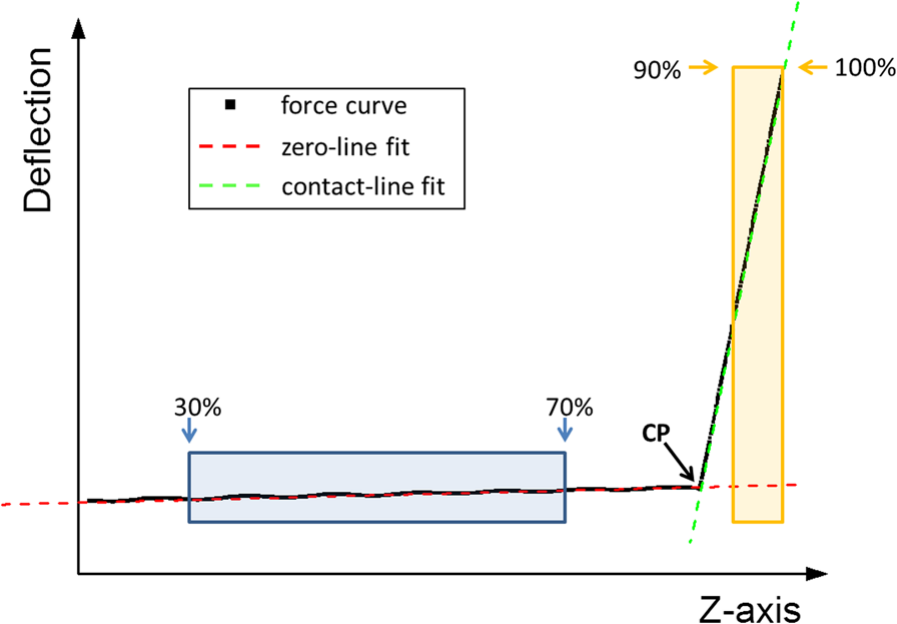
Example of the use of the routine fit intersection. The zero-line fit (red line) is performed in the interval 10–70% of the curve (blue-highlighted section) while the contact-line fit (green line) is performed in the interval 90–100% of the curve (yellow-highlighted section). The black arrow identifies the contact point (CP)

- *Selection criteria*: provides two different ways to select the four FCs on which the *Optimizer* routine can operate to determine the best analysis parameters. The default option is “*random*”, i.e. the four FCs will be randomly selected, while, by choosing the “*insert position*” option, the user can select the X and Y coordinates of the desired curves to be optimized.
- *Percentage of curve used for the zeroline subtraction*: allows selecting the FC section that will be linearly fitted to determine the zeroline background. The option requires inserting a percentage of the curve: 0% is the starting point (i.e. the farthest from the surface) while 100% represents the point of maximum applied load. An example is reported in Fig. 2.
- *Contact point identification*: The contact point (CP) is a crucial parameter to perform a correct calculation of the physical information contained in the contact area of each FC. To account for the various experimental conditions and FC forms, we have introduced three different routines to determine the CP:
  (i) *Derivative + smooth*: this routine operates by performing a moving average smoothing of the force curve (with the number of smoothing points set by the user), followed by a derivative of the force curve and by a smoothing of the derivative curve. Finally, the routine identifies as contact point the first zero value encountered on the smoothed derivative curve, from the point of maximum load. This routine is particularly effective in case of noisy data.
  (ii) *Lowest value + smooth*: this routine operates by performing a moving average smoothing of the force curve (with the number of smoothing points chosen by the user); next, it identifies the contact point as the first minimum encountered starting from the point of maximum load. This routine is particularly suitable for force curves with low noise.
  (iii) *Fit intersection*: this routine performs a linear fit of the contact line (whose first and last point can be set by the user, expressed in percentage of the whole force curve). Then, the contact point is identified as the closest to the intersection between the contact-line fit and the previously calculated zero-line fit, as shown in Fig. 3. This routine is particularly suitable for force curves acquired on hard samples.

**Fig. 4:**
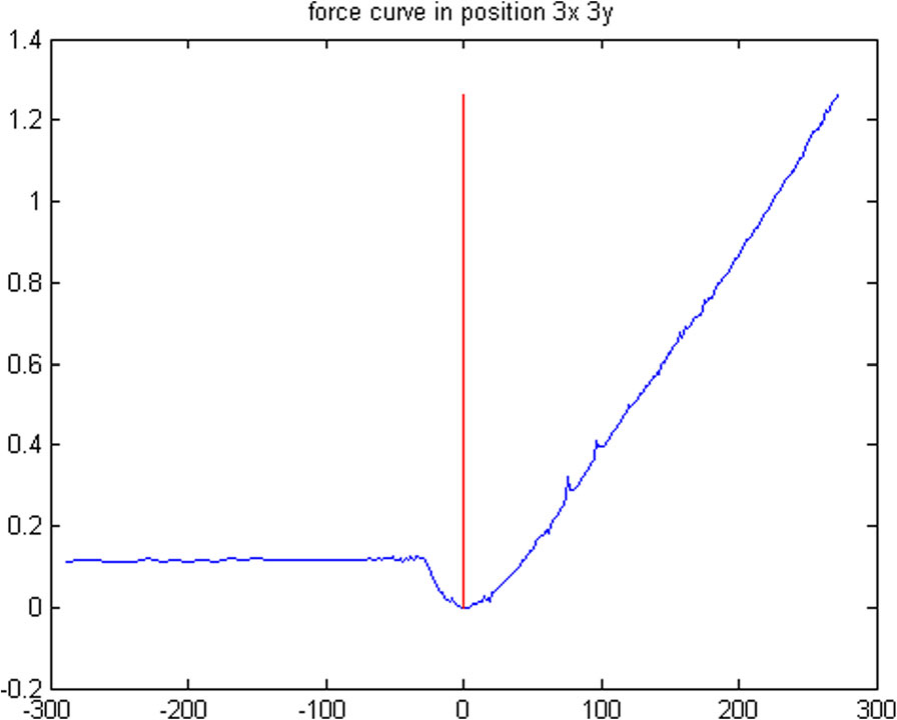
How *FC_Analysis* visualizes the identified Contact Point to grant the user’s visual inspection: the identified contact point is located at the intersection between the red vertical line and the force curve

In all cases, the software marks the identified contact point through a vertical red line, as shown in Fig. 4. Typically, a user visual check-up is required to determine the effectiveness of the chosen routine in the CP identification. In the case of FV images, the Z-axis coordinate of the contact point is stored and will be used to build the zero-force, topographical, image of the sample.

### Programs

The software package *FC_analysis* contains 4 different routines to analyse the contact part of the FCs, in order to extract the nanomechanical information: *Optimizer*, *Multicontact*, *Multicurve* and *Multindentation*.

#### Optimizer

**Fig. 5:**
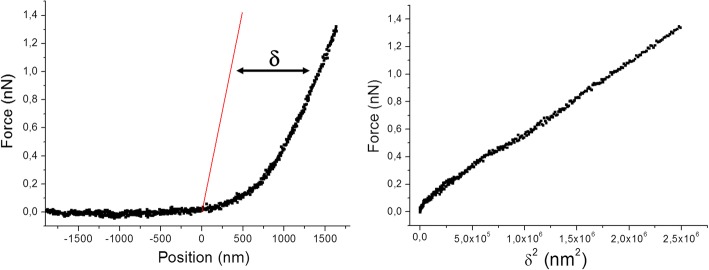
Indentation depth and indentation curve: (left) example of calculation of the indentation depth (δ): the red line represents the FC obtained on an incompressible substrate while the black one is the FC under analysis; (right) correspondent indentation curve

This routine aims to perform a fast screening of the force curve map in order to determine rapidly the best parameters to be used in the analysis of all the FCs. Indeed, this section of the software selects 4 force curves, following the *selection criteria* chosen by the user, and should be used at the beginning of the image analysis, in order to save time in the following thorough calculations. Operatively, the software visualizes, for each FC, the steps that lead to the calculation of the indentation curve (defined as the force versus the square power of the indentation depth): the zeroline subtraction, the CP identification and the calculation of the indentation depth (δ). The indentation depth is defined as the difference between the contact line of the actual force curve and the line that results from the calibration of the detector’s sensitivity. An example of how δ is calculated is reported in Fig. 5.

#### Multicontact

This routine applies the zeroline subtraction and the *contact point identification* to each FCs in the whole map (or just a portion of it, if the *partial selection* is highlighted); then, it performs a linear fit of the resulting contact line. The user can choose the percentage of contact line used for the fit: the default interval is 0–100%, where 0% indicates the contact point and 100% the point of maximum load.

This routine produces two outputs: a topography map and a stiffness map. The topography map is composed by the heights of the sample determined from the position of the contact points of each FC. The stiffness map contains, in each position, the value of the slope of the contact line, obtained through the linear fit described above. Both maps are saved automatically as jpg files. The stiffness map is also saved as a text file with file-name chosen by the user, and contains the matrix of the calculated values. In this text file, the first row and the first column contain, respectively, the X and Y position, allowing a further processing of the data obtained. This routine is particularly suitable for experiments involving hard un-deformable samples.

#### Multicurve

This routine applies the whole set of parameters and subroutines, selected through the *Optimizer*, to the whole set of FCs (or just a portion of them, if the “*partial selection*” is highlighted), and produces three outputs: a topography map obtained from the positions of the CPs, a complete set of indentation curves and a preliminary Young’s modulus map.

For each FC, *Multicurve* generates the corresponding indentation curve that is automatically saved as a separate text file, with the name “ind_posx_posy.txt”, that can be further refined using the *Multindentation* routine. The preliminary Young’s modulus map is based on a non-optimized linear fit of the indentation curve and the values are reported in kPa. This map provides just a first view of the analysis result and it is saved automatically as jpeg file and as txt file. The use of *Multicurve* is especially recommended for applications involving soft samples such as most biological specimens.

#### Multindentation

**Fig. 6:**
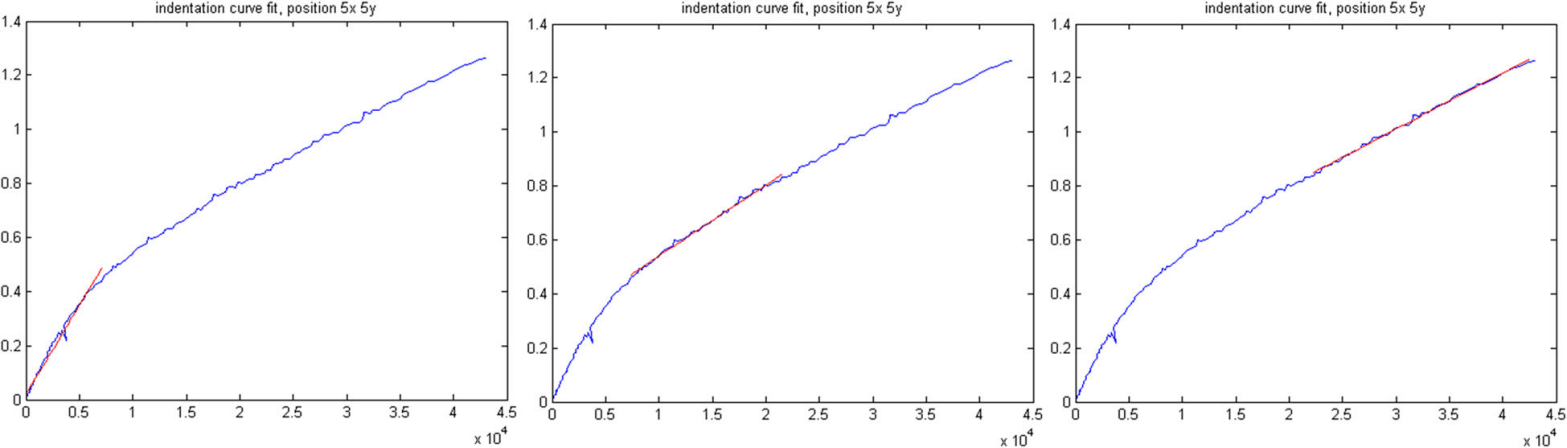
example of application of the force tomography, from left to right different runs of the *Multindentation* program with different selected indentation curve sections to be fitted: 0–35%, 40–75% and 80–100%

This algorithm analyses the indentation curves previously generated with the *Multicurve* subroutine. This fast fitting routine performs a linear fit of a selected percentage of the indentation curve, where 0% indicates the contact point and 100% the point of maximum load. The output is a quantitative map of Young’s modulus (E), the detailed calculations are reported in the next paragraph. In *Multindentation* the user can select the units in which the obtained Young’s modulus values will be expressed (kPa, MPa or GPa) and can customize the E calculation according to the experimental conditions, by inserting the values of ν, α and C_0_ (see Eq. 3). The quantitative Young’s modulus map is saved as a jpg file and as a text file, which contains the matrix of the calculated values. In this file, the first row and first column contain, respectively, the X and Y position. It should be noted that, by using this fast routine, the user can perform analyses using the so-called force tomography mode [12, 13], namely, the investigation of the Young’s modulus of deep stiff areas buried underneath the sample’s surface through fitting of different portions of a force curve. This is possible by using the routine several times while changing the percentage of the indentation curves to be fitted. An example of such application is reported in Fig. 6 where we show, in red, the different slopes obtained by analysing different portions of a given FC, corresponding to different penetration depths of the tip. For each run, the routine generates a new Young’s modulus map, whose name will explicitly show the selected extremities of the fit region, ensuring a fast and direct comparison of the elasticity behaviour of the sample at different indentation depths.

### Equations for the calculation of the Young’s modulus

In the analysis of the indentation curves, we have used a theoretical model based on the Hertz theory [14] modified by Sneddon [15] and Bilodeau [16] for the case of conical and pyramidal tip respectively. The evaluation of spherical tips, as well as other fitting protocols will be implemented soon. To evaluate the resulting Young’s modulus (E), the indentation curve is fitted using the Eq. (2):

2 $$ F={C}_0\frac{2E{\delta}^2}{\pi \left(1-{v}^2\right)\tan \alpha } $$

Where F is the applied load (expressed in nN), δ is the indentation depth (expressed in nm), ν is the Poisson ratio, α is the tip’s opening half angle and C_0_ is the Bilodeau coefficient (equal to 1 if the tip is conical and 1.46 if the tip shape is a four-side pyramid). The value of the Young’s modulus results from the slope (B) of the linear fit of the indentation curve through the formula (3):

3 $$ E=\frac{3\pi B\tan \alpha }{8{C}_0} $$

### Working with the data: how to export the maps in a third party analysis software

The three routines, *Multicontact*, *Multicurve* and *Multindentation*, automatically export their maps as jpeg files with a name that contains the map name and some analysis specifications (partial or total map, units and range used for the fit). Furthermore, the data are also stored into a text file for subsequent processing or reprocessing with other analysis software (for instance to remove outline points). The text file, whose name is chosen by the user, is composed by an array of data in which the first row and the first column contain, respectively, the X and Y position of the analysed FC. In this framework, it is useful to describe how the data generated by *FC_analysis* can be imported in the widely used free software Gwyddion (Gwyddion http://www.gwyddion.net): (i) uncheck the box for automatic recognition; (ii) select the option “*raw data file*”; (iii) input the number of pixels that compose the image (i.e. the number of FCs x row) and the real physical dimensions of the image; (iv) in the “*data format*” sheet select “*text data*” (instead of binary), insert # 1 in the field “*start from line*” and insert # 1 in the field “*each row skip*” (v) the field delimiter is a whitespace.

## Materials and methods

### Specimens’ preparation

To test the capabilities of the software package, we investigated three different classes of samples: (i) we calculated the Young’s modulus map of a peculiar structure of the human Red Blood Cell (RBCs) membrane; (ii) we performed a stiffness map on a sample consisting in a tissue from the digestive gland of a mussel embedded in epoxy resin; (iii) we determined the stiffness of metallic inclusions observed in a meteorite section.

(i) We collected the RBCs from a healthy donor, after informed written consent. The samples were collected for the sole purpose of this study and of previously published studies [17, 18]. The cells were prepared by smearing, onto commercial poly-l-lysine coated glass slides, a purified suspension of RBCs after several days of accelerated in vitro ageing (in starvation condition). The rational of this approach was that at increasing ageing time the presence of different peculiar defects can be observed on the cell membrane. The smears were performed and were air-dried at room temperature. A detailed description of the sample preparation as well as an introduction to the RBCs and their ageing can be found in previously published papers [17, 18].
(ii) Sections of the digestive gland of a mussel were embedded in epoxy resin and cut in approximately 250 nm-thick tissue slices using an ultramicrotome. The collection of the mussels and the protocol for the inclusion and preparation are described in detail elsewhere [19].
(iii) The approximately 50 μm thick meteorite section was obtained from a full meteorite fragment, polished with 1 μm diamond paste and analysed. More detail about the preparation of this specimen can be found in previously published work [20–22].

### Atomic force microscopy

The FV maps were acquired with two different microscopes, using different setups: a homemade AFM, described in detail elsewhere [23, 24], and a commercial AFM (FlexAFM, Nanosurf, Liestal, CH). The homemade AFM works in the “row by row” acquisition modality and, to perform a FV map, requires the collection of a topography image followed by the collection of the series of FCs. The FCs generated are directly saved as separated *.txt files, each containing a single complete force curve. For the experiments using this microscope, we chose silicon nitride cantilevers from Bruker MSCT probes (Camarillo, CA, USA) with a 4-sided pyramidal tip shape and an elastic constant of 0,02 N/m. The mechanical properties of the cantilever were determined using the thermal calibration method [25]. The FlexAFM works in the “raster” scan acquisition modality and collects directly the entire FV map. The map is saved as a single *.nid file, and each single FC can be extracted in separated *.txt files through a dedicated free software, developed by the Nanosurf. In this case, we used silicon nitride Budget Sensors TapAl-G probes (Innovative Sensors, Sofia, BG) with a 4-sided pyramidal tip shape and thermally calibrated elastic constant of values comprised between 15 and 17 N/m. To evaluate the time needed to perform a complete data analysis we refer to our test workstation, a desktop PC with Intel® Core ™ i7–3770 CPU @ 3.40 GHz, 8 GB of RAM, running win7 64-bit. With this hardware configuration, we processed ten thousand FCs, i.e. a 100 × 100 map, in about 3 min through *Multicurve*, in about 2 min with *Multicontact* and *Multindentation*. Naturally, we foresee the possibility of upgrading the software with optimized routines for multicore processors and to exploit the GPU calculation power.

## Results and discussion

**Fig. 7:**
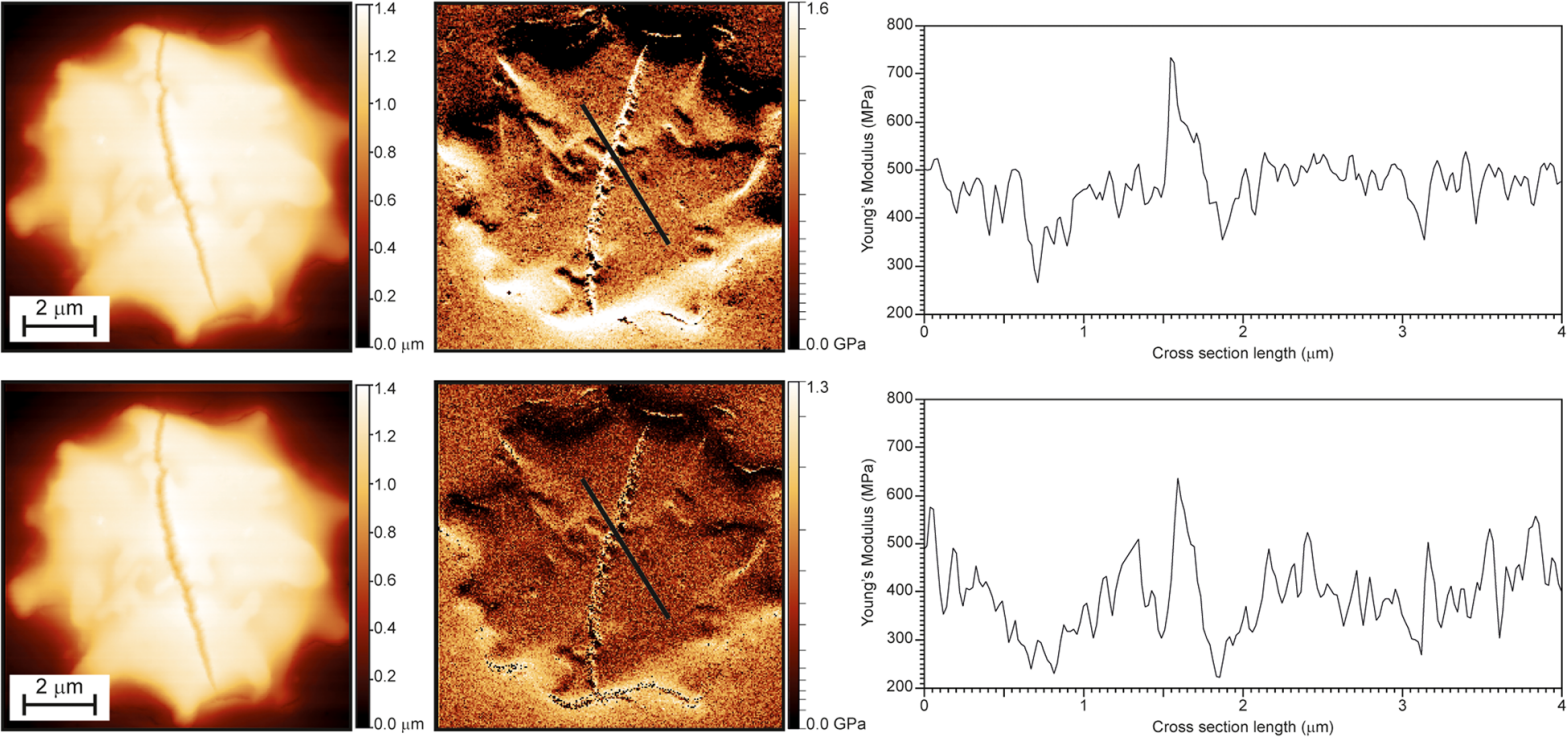
comparison between *FC_analysis* and the JPK analysis software, topography (left) and elasticity maps (center) calculated using the JPK analysis software (top panels) or the *FC_analysis* (bottom panels). The analyses were performed on the very same 256 × 256 FV image acquired with a Nanowizard III microscope. The right panels show two cross-sections of the Young modulus maps collected on the two images (right)

To demonstrate that *FC_analysis* can be used to analyse the force curve maps obtained from different AFMs, with radically diverse instrumental setups, analysis parameters, modalities and sample’s characteristics, we performed experiments on two different microscopes on samples ranging from materials sciences to biology. For each test case, we performed a manual validation of at least 200 randomly selected force curves. In addition, to validate the *FC_analysis* results and to ensure their reliability, we collected a force volume image using a Nanowizard III microscope (JPK instruments, Berlin, DE; courtesy of prof. Dietler, LPMV – EPFL, Lausanne). We analysed the curves using the dedicated JPK analysis software and compared the results with those obtained using FC_analysis. For this control experiment we considered human erythrocytes (RBCs). These cells play the essential physiological role of oxygen carriers to the tissues, their biochemical pathways are relatively simple, their structural architecture is based on the properties of their membrane-skeleton and they are cheap and easy to purify and manipulate. Indeed, despite its robustness, the membrane-skeleton is also extremely flexible and is responsible for the erythrocytes’ mechanical properties and their ability to change dynamically shape in the bloodstream. It consists of a dense network of tetrameric polymers of spectrin connected with the lipid double-layer through proteic “junctional complexes” and ankirins [26]. A failure of this structure, due to pathologies or cell ageing, impairs the shape preservation of erythrocytes and their functionality. In addition to the change of local mechanical properties, the erythrocyte’s aging pathway can lead to morphological alterations of the cell, with the appearance of a typical morphological phenotype knows as echinocyte. We collected an image of a single aged erythrocyte that presented fully developed alterations, using the Nanowizard III microscope in force volume mode (256 × 256 FCs). We analysed the resulting force curve map using the JPK data analysis software and extracted each force curve in text mode to allow the transfer of the curves to the *FC_analysis* software. We calculated the mechanical and topographical properties of the sample using the same calculation parameters for the two software: K_C_ = 0,164 N/m, S = 0,025 V/nm (which translates to 40,00 nm/V for the JPK software), Z-stage sensitivity 10^9^ nm/step, zeroline subtraction fit from 0 to 70% (30 to 100% for the JPK software), “lowest value + smooth” contact point identification routine with smoothing interval of 3 points, Poisson Ratio of 0,5, 4-sided pyramidal tip shape with 23,5° tip half angle (half angle to edge for the JPK software), and percentage of the indentation curve fitted 0–100%. The results, shown in detail in Fig. 7, and in particular the cross-sections reported in the right panels, demonstrate that the *FC_analysis* produces topography and Young’s modulus maps that are perfectly compatible with those obtained with the commercial software.

Once we have demonstrated the reliability of the *FC_analysis* calculations, we exploited its capabilities by studying three test cases. In the first, we considered a peculiar morphological defect of erythrocytes that arises during their aging pathway: the appearance of peeled areas, where the cell membrane has detached from the underlying skeleton, directly exposing this structure. During the aging, the covalent protein bridges between these two structures can become weaker and the unsupported patches of the membrane become, at least transiently, floating. In these conditions, the application of a physical stress (such as the air-drying process) can cause the removal of a floating patch of membrane, exposing the virtually intact cell skeleton. This peculiar morphological defect provides a very interesting way to directly visualize and investigate the mechanical characteristics and the architecture of the underlying membrane-skeleton without the “disturb” induced by the lipid bilayer.

**Fig. 8:**
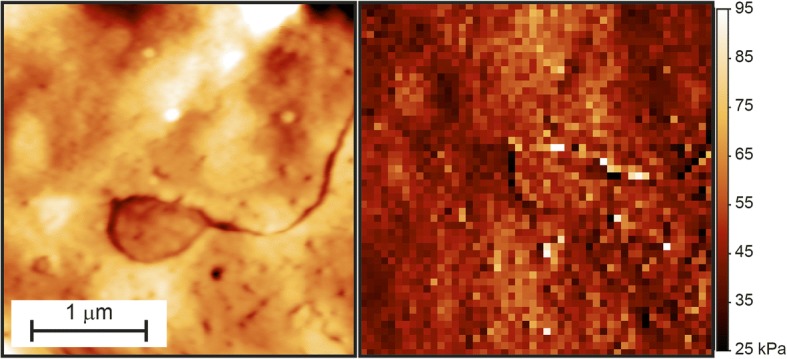
FC map acquired onto a peeling-bearing RBC: topographical (500 × 500 pts) image and corresponding (50 × 50 pts) elasticity map acquired onto the surface of a red blood cell with a “peeling” area. Data collected with the homemade AFM

The membrane of a peeled RBC is shown in Fig. 8, as imaged by the homemade AFM. A missing patch of lipid bilayer is clearly visible in the 3μmX3μm topography image (500 × 500 points) while the Young’s modulus map (50X50 FCs) generated by the software highlights how the elasticity, measured inside and outside the peeling area, is the same. This analysis was carried out by a two-step procedure. First the *Multicurve* program was used with the following configuration: K_C_ = 0,02 N/m, S = 0,0013 V/nm, Z-stage sensitivity 0,0643 nm/step, zeroline subtraction fit from 0 to 60%, “derivative + smooth” contact point identification routine with smoothing interval of 5 points. Subsequently the indentation curves generated by *Multicurve* was analysed using *Multindentation* with Poisson Ratio of 0,5, tip’s half angle 23,5°, 4-syded pyramidal tip shape and percentage of the indentation curve fitted 10–60%.

This data clearly suggests that the peeling process does not affect the whole membrane-skeleton but involves only the lipid bilayer. The exposed cytoskeleton, indeed, maintains the same elasticity characteristics of the other parts of the cell and provides the dominant contributor to the measured value [27].

**Fig. 9:**
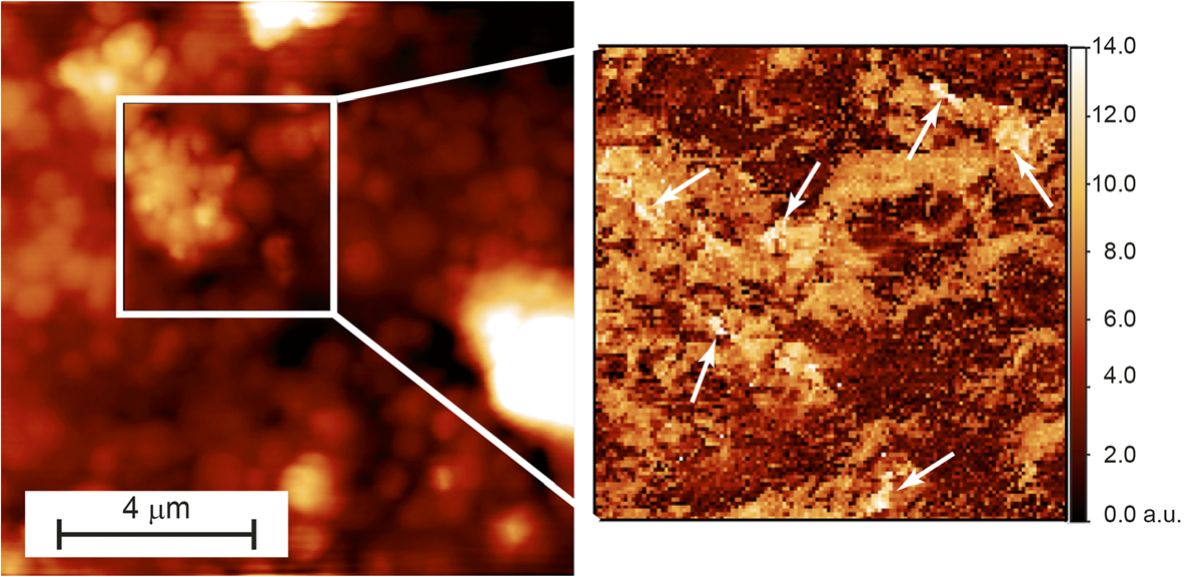
FC map acquired onto a section of mussel’s glands, embedded in epoxy resin: topographical (512 × 512 pts) image and corresponding (128 × 128 pts) stiffness map acquired in the highlighted region. The white arrows indicates areas with very high local stiffness, which suggests the presence of non-biological material. Data collected with the FlexAFM

As a second test case we exploited the capability of the software in the analysis of thin sections of biological tissues. We studied mussels (*Mytilus galloprovincialis*), which are non-motile organisms that feed by filtering the surrounding water. This means that all undigested material tends to accumulate in the animal’s organs and, including in their digestive glands. A high-resolution identification of stiffer exogenous nanostructured material in these organs and the evaluation of their effect on the structure of the tissue are extremely useful parameters to determine the sea-water pollution levels. In the left panel of Fig. 9 we show a 10μmX10μm AFM topography image, collected with the FlexAFM, of one of the tissue slices while, in the right panel, we report a 128 × 128 stiffness map measured on the highlighted 4μmX4μm image subsection and post processed through the *FC_analysis*. This analysis was carried out using the *Multicontact* program with the following configuration: K_C_ = 15,72 N/m, S = 0,00789 V/nm, Z-stage sensitivity 10^9^, zeroline subtraction fit from 10 to 80%, “lowest value + smooth” contact point identification routine with smoothing interval of 5 points and percentage of the contact line to fit from 0 to 100%.

We chose this region for the FC mapping because of the large number of little secretory granules observed. Since the analysed tissue samples were embedded in epoxy resin, we expected a homogeneous stiffness map. Remarkably, the results show a very inhomogeneous landscape. The brighter (stiffer) areas are correlated in the corresponding topography with the secretive granules, while the darker (softer) areas seem to be correlated with the secretory ducts that surround these structures. Moreover, there are several very bright small areas, indicated by the white arrows in the image, which suggest the presence of material of non-biological nature. Since these brighter spots correspond to no features in the morphological image, this could indicate that the exogenous material is embedded in the tissue. This data highlights the capabilities of the FV analysis and show how the *FC_analysis* can be used to extract additional, non-morphological, information from biological tissues.

**Fig. 10:**
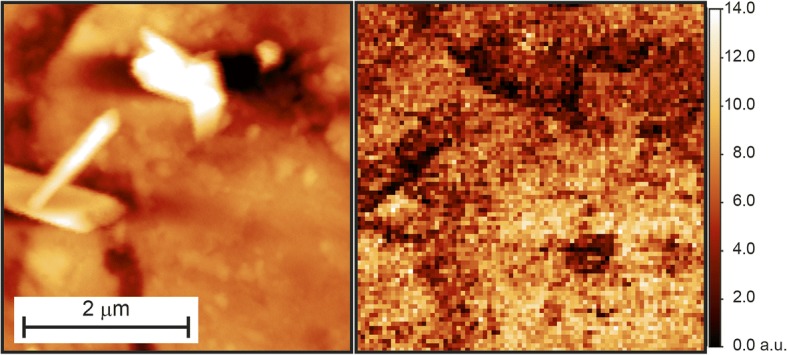
FC map acquired onto a thin section of meteorite: topographical (512 × 512 pts) image and corresponding (80 × 80 pts) stiffness map acquired in the same 4 μm region. Data collected with the FlexAFM

As a third test case, we applied the software to the analysis of FV maps acquired on an ordinary chondrite, representing the most abundant population of meteorites. This is a typical hard material, and is mainly composed of a matrix of olivine and pyroxene, two silica minerals, in which metallic inclusions of iron (generally in martensitic form) can be found. The presence and characteristics of these inclusions is believed to be strongly influenced by the space weathering phenomenon [28]. From a structural point of view, the martensitic clusters are stiffer than the surrounding matrix and a nanomechanical mapping of these samples can reveal presence, dimensions and distribution of metallic inclusions that can’t be distinguished by means of simple topographical images. To perform such a study we use the FlexAFM. Figure 10 shows a topographical image (4 × 4 μm) obtained in tapping mode (512 points per line) and the corresponding stiffness map (80 force curves per line). The analysis was carried out using the *Multicontact* program with the following parameters: K_C_ = 15,66 N/m, S = 0,00789 V/nm, Z-stage sensitivity 10^9^, zeroline subtraction fit from 10 to 85%, “lowest value + smooth” contact point identification routine with smoothing interval of 5 points and percentage of the contact line to fit from 0 to 100%.

The topographical image (Fig. 10, left panel) evidences a rough surface with some outlining features, which correspond, in the stiffness map (right panel), to darker areas. On the other hand, the lower right section of the image appears flatter and nanostructured, and this corresponds to lighter areas of the stiffness map. This is consistent with previous studies that associate the nanostructuring of metallic surfaces to the formation of martensitic areas, which are stiffer than the non-nanostructured zones [20, 21].

## Conclusions

We have presented the software package *FC_analysis*, a new, simple and versatile software that aims to support the analysis of AFM force curves and force volume maps, obtained with a large variety of AFMs (homemade or commercially available). In particular, to our best knowledge, there is no freely available dedicated FC analysis software for simple *.txt files and, under this point of view, it is different from other GPL or commercial software, such as OpenFovea or SPIP (http://www.imagemet.com/), that are dedicated to the analysis of force volume images from commercial AFMs.

A novelty of *FC_analysis* resides in its high flexibility, provided by the direct and immediate control of each parameter related to the setup, such as: cantilever’s elastic constant, detector sensitivity, tip’s shape and scanning modality. This guarantees a high applicability to different instrumental setups and experimental conditions. Furthermore, the *Optimizer* routine allows an immediate visual control of the effect of every single parameter on the analysis of the force curves, thus allowing the rapid optimization of the whole procedure. This ensures that this software is user friendly and completely customizable.

Another major advantage of this software package resides in its ability to analyse rectangular portions of FV maps, allowing the optimization of the analysis parameters for each area of an image and favouring a correct determination of the Young’s modulus even from inhomogeneous samples. This options results interesting also for the analysis of partially acquired maps, i.e. maps that during the acquisition were interrupted.

In this work we have shown how *FC_analysis* can be applied to the study of hard, soft and inhomogeneous samples. For the hard, non-deformable specimens, the dedicated *Multicontact* routine provides the stiffness and topography map. For the soft, deformable, samples such as the majority of biological specimens, the two dedicated routines, *Multicurve* and *Multindentation*, should be used to study the elasticity of the samples as function of the indentation. These two routines differ in the interval of the FC fitted allowing a fine-tuning in the determination of the correct elasticity of these particularly challenging samples.

To confirm the capabilities and the versatility of *FC_analysis*, we demonstrated its use with images and maps collected with two different AFMs working in two different experimental setups and we characterized three different specimens: a single air-dried erythrocyte, a tissue embedded in epoxy resin and a hard section of a meteorite.

In conclusion, the *FC_analysis* software package can lead to a semi-automatized analytical approach to the study of FCs and FV maps, with a direct control of each parameter involved in the calculation. Moreover it can be used to perform force tomography analyses and to work with maps obtained on very inhomogeneous samples. In our opinion, this software can be a valuable aid for the rapid analysis of FV maps regardless of the AFM instrument used for their acquisition.

For the future development of the software package, the user’s feedback will be kept in great consideration. In particular, we’ll focus our efforts in the implementation of more sophisticated routines to identify the contact point and in the implementation of additional options, such as the analytical modelling of spherical tips and the introduction of different fitting strategies. Also, we will make efforts to extend the use of *FC_analysis* to data collected with different commercial AFMs, especially in cases where there is no freely available analysis software.

## Availability and requirements

**Project name**: *FC_analysis.*

**Software executable file**: FC_Analysis_v3.exe submitted as Additional file 1

**Operating systems**: Windows

**Programming language**: Matlab

**Other requirements**: Matlab Compiler Runtime (MCR) (version 8.0 or newer) that can be freely downloaded from the Mathworks website: http://uk.mathworks.com/products/compiler/mcr

**License**: free

**Any restriction to use by non-academics**: none

## Additional file

Additional file 1: FC_analysis executable file. (EXE 498 kb)

## Abbreviations

AFM: Atomic Force Microscope
CP: Contact point
FC: Force curve
FV: Force volume
RBC: Red blood cells
